# Measuring physical and mental health during pregnancy and postpartum in an Australian childbearing population - validation of the PROMIS Global Short Form

**DOI:** 10.1186/s12884-019-2546-6

**Published:** 2019-10-22

**Authors:** Valerie Slavin, Jenny Gamble, Debra K. Creedy, Jennifer Fenwick, Julie Pallant

**Affiliations:** 1 School of Nursing & Midwifery, Griffith University, Logan Campus, University Drive, Meadowbrook, QLD 4131 Australia; 2 Gold Coast University Hospital, 1 Hospital Boulevard, Southport, QLD 4215 Australia

**Keywords:** Patient Reported Outcomes Measurement Information System (PROMIS), Rasch analysis, Psychometric evaluation, Pregnant women, Postpartum, International Consortium for Health Outcomes Measurement (ICHOM), Health related quality of life (HRQoL), Quality of life, Mental health, Physical health

## Abstract

**Background:**

Health related quality of life is a critical concept during the perinatal period but remains under-researched. The International Consortium for Health Outcomes Measurement have included the Patient Reported Outcomes Measurement Information System (PROMIS®) Global Short Form (GSF) in their core outcome set for pregnancy and childbirth to measure health related quality of life. The PROMIS GSF has not been fully evaluated as a valid and reliable instrument in this population. This study assessed the psychometric properties of the PROMIS GSF during pregnancy and postpartum period.

**Methods:**

PROMIS GSF was administered to a sample of 309 pregnant women at four time-points during pregnancy (≤ 27 and 36-weeks) and postpartum (6- and 26-weeks). The structural validity, internal consistency reliability, construct validity, and responsiveness of the PROMIS GSF were evaluated. The internal structure of the PROMIS GSF was explored using Rasch Measurement Theory. Response format, item fit, differential item functioning (item bias), dimensionality of the scale and its targeting were assessed.

**Results:**

Two revised subscales (Mental Health: four items; and Physical Health: five items) showed good fit to the Rasch model. The revised mental health subscale demonstrated good internal consistency reliability during pregnancy and postpartum period (α = .88 and .87, respectively). The internal consistency reliability of the physical health subscale was adequate (α = .76 and .75, respectively). The revised mental health subscale was sensitive to group differences according to a history of mental health disorder, income, smoking status, drug use, stress levels and planned versus unplanned pregnancy. Differences in scores on the revised physical subscale were detected for groups based on obesity, income, drug use, smoking status, stress, and history of mental health disorders. Scores on both subscales recorded significant changes across the four time-points, spanning pregnancy and postpartum period.

**Conclusions:**

The revised version of the PROMIS GSF was better able to measure mental and physical health during pregnancy and postpartum period compared to the original version. Findings support the clinical and research application of the PROMIS GSF within the International Consortium for Health Outcomes Measurement Standard Set of Outcome Measures for Pregnancy and Childbirth. Ongoing psychometric analysis of the PROMIS GSF is recommended in other maternity populations.

## Background

Health related quality of life (HRQoL) is well researched in areas of disease and chronic health conditions but remains under-researched in women’s perinatal health. While HRQoL is not generally well defined within the literature, it does refer to a multi-dimensional concept to examine the impact of health status on quality of life [1]. Adapting a definition by O’Connor [2], HRQoL for maternity populations can be defined as ‘*a multi-dimensional concept referring to a woman’s perception of the influence of her pregnancy, birth and postpartum condition, her care provision and any intervention and treatment on her physical, mental, emotional, and social functioning*. HRQoL is acknowledged as a critical concept in the childbearing period [3] that goes beyond the traditional broad metrics of morbidity, mortality and life expectancy. While several socio-demographic, physical and psychological factors are known to influence the quality of life of pregnant women [4], the maternity model of care women receive during this time may also have a significant impact on their future HRQoL.

Around the world women are experiencing fragmented maternity care [5], and unnecessary medical procedures and birth interventions [6]. Cesarean birth rates are at an all-time high with a continued upward trend for cesarean birth in all but a few OECD (Organization for Economic Cooperation and Development) countries. In countries with the highest rates of cesarean birth, (for example Turkey, Mexico and Chile), around half of all babies are born this way [7]. In Australia, where the current study was conducted, one in three women experienced surgical birth in 2016 [8]. A growing number of women report obstetric violence; bullying, abuse, disrespect and coercion at the hands of their care givers [9–11]. Birth fear and birth related trauma are increasingly reported in the literature [12, 13].

While such events during pregnancy, birth and the postpartum period may adversely affect HRQoL, little is known. This may reflect the challenges of measuring such a construct during a dynamic period in women’s lives. Furthermore, a lack of validated tools for use during this unique time, and a wide variation in outcomes measured, compounds the issue. A systematic review of quality of life measures utilized in pregnant and postpartum mothers revealed not only a significant variation in both outcomes and outcome measures used, but a limited number of woman-reported outcome measures that had been formally developed and validated with maternity populations [14]. The most widely-used scales were the SF-36 [15] and SF-12 [16] from the Medical Outcomes Study (MOS), generic instruments that measures HRQoL in diverse patient populations. Most included studies focused on the psychometric properties of the measurement tool alone, and all failed to explore whether the measures were missing relevant items and constructs. The extent to which quality of life items had similar meaning to pregnant and postpartum women compared to non-pregnant women has not been investigated previously. Such limitations can contribute to poor clinical practice or policy change decisions based on inaccurate findings.

To address the heterogeneity of measures and outcomes often used in research, there is a growing consensus advocating the standardization of outcomes and outcome measurement using core outcome sets to improve the synthesis of research findings and minimize research wastage [14, 17]. In 2016, the International Consortium for Health Outcomes Measurement (ICHOM) published a core outcome set for use during pregnancy and childbirth [18]. This minimum standard set of internationally appropriate outcome measures is recommended to be collected during pregnancy and postpartum period to assist health providers evaluate and improve the value of care provision [19]. Acknowledging the value of measuring HRQoL, ICHOM included the Patient Reported Outcomes Measurement Information System (PROMIS®) Global Short Form (PROMIS GSF).

Using global health items from an item bank developed by the National Institute of Health, Hays and colleagues [20] validated the 10-item PROMIS GSF using classic test theory and a sample drawn from the 2000 United States (US) census data. Classic test theory has some weaknesses compared to contemporary techniques, particularly in the approach to data management and analysis [21]. In classic test theory the approach is to describe a full data set, whereas, techniques such as Rasch Measurement Theory obtain data that fits the model, thereby overcoming the limitations of classic test theory and enhancing the precision of outcome measures [22]. Modern test theory, including Rasch models can improve on the classical approach when validating woman-reported outcome measures [23]. The sample used by Hays et al. [20] included both clinical and community samples of women (52%) and men (48%) with a mean age of 53 years (range 18–100). ICHOM working party members recognized the limited use of some of the included measures in maternity populations as a limitation [19]. A HRQoL instrument must be valid and have high reliability and responsiveness in the population being studied [24]. To facilitate the universal adoption of the ICHOM Standard Set into all trials evaluating maternity care, there is an urgent need to evaluate the tool with maternity populations. To date, only one paper has reported using the PROMIS GSF with women during pregnancy [25]. The findings of this validation study are limited however, by the culturally diverse background of the 161 pregnant women in the United States: Hispanic (42%), non-Hispanic woman of colour (37%), non-Hispanic white (14%), and multiracial/other (7%), limited evaluation of structural validity, and no evaluation during the postpartum period [25]. To address these limitations and facilitate ongoing and global comparison of outcomes, the current study sought to conduct a Rasch-based psychometric evaluation of the PROMIS GSF to measure HRQoL in one Australian childbearing sample using a standardized approach.

## Aims

The MoMeNT Study (Models Meeting Needs Over Time) is a longitudinal cohort study with two primary aims: (1) to assess the feasibility of the ICHOM Standard Set of Outcome Measures for Pregnancy and Childbirth in one Australian population, and (2) report the perinatal outcomes for women accessing all models of maternity care in one facility to facilitate benchmarking and comparison of maternity models of care.

## Methods

A prospective, observational cohort study was conducted.

### Setting

The study was conducted in one large tertiary referral hospital in a metropolitan area of Queensland, Australia. The study site provides two broad models of maternity care: caseload midwifery care and non-caseload care. Caseload midwifery care refers to one primary midwife who is responsible for the care of a caseload of approximately 40 women per year, and provides holistic, relational continuity of care for each woman during pregnancy, labour, birth and for up to six-weeks postpartum with back-up from a midwife partner. Caseload midwives can provide pregnancy care in the home, satellite clinics and the hospital. Labour and birth care is provided in the hospital setting. Non-caseload care is an umbrella term encompassing midwifery, general practitioner, obstetric care or a combination. Women receiving non-caseload care receive no continuity of care. Labour and birth care is generally provided by carers not usually known by the woman. Postpartum care by a midwife is limited (usually two visits).

### Participants, sample size, recruitment and attrition

Pregnant women were eligible to participate if they were 27-weeks gestation or less (at recruitment), aged 18 years or more, and able to complete an online survey in English. Women with an existing serious mental illness under the care of a psychiatrist were excluded. Recruitment occurred between August 2017 and March 2018.

Sample size was based on evaluating the broad effect of model of maternity care on maternal health and wellbeing. G*Power (3.0.10) sample size calculator was used. To identify a mean difference (two-tail) with a 50% effect size, 5% estimated error and 95% power (1 – β err prob), 210 participants were required. To allow for 20% attrition 252 participants were needed (126 in each group). Of the 1275 women screened in the public caseload midwifery and non-caseload models, 528 (41.4%) were eligible to participate. Reasons for ineligibility were due to: greater gestation date (*n* = 723), unable to communicate in English (*n* = 20), age less than 18 years (*n* = 2), and mental health disorder under the care of psychiatrist (*n* = 2). Eligibility was care model dependent: 25.8% of women in non-caseload and 85.7% of women in caseload were eligible to participate. Of those eligible, 354 women (67%) were invited to participate. In non-caseload care 20 women were missed. In the caseload midwifery model 108 women (40.1%) were not initially invited by their primary midwife. Feedback from midwives suggested barriers to recruitment included having no tablet device or consent forms available in the home setting. Of those invited, 336 (94.9%) completed consent forms. Reasons for declining were: too busy (*n* = 8), intended to move during pregnancy (*n* = 2), or no reason given (*n* = 8). The baseline survey was completed by 309 (92.0%) women.

**Fig. 1:**
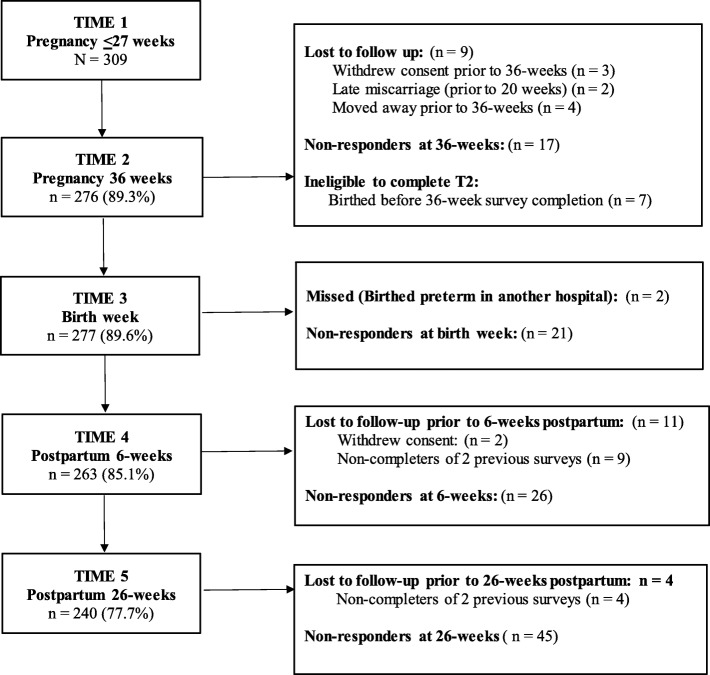
Flow of participants from pregnancy to 26 weeks postpartum

Participants birthed between November 2017 and September 2018. Of the 309 women who completed the baseline survey (Time 1), nine did not continue (as outlined in the participant flow diagram - Fig. 1). Three women withdrew consent, two experienced late miscarriage, and four moved away. Eight women were ineligible to complete the 36-week survey due to birthing prematurely/prior to 36-weeks, six birthed in another hospital, and two were missed. These women remained in the study and received subsequent surveys.

### Measures

The ICHOM core outcome set was administered in full according to the ICHOM Pregnancy and Childbirth Data Collection Reference Guide [18]. Each online survey included the PROMIS GSF. The baseline survey obtained socio-demographic, medical, obstetric and psychosocial details including participant age, gestation, relationship status, education, income and country of birth (See Additional file 1 for details of all variables used in analysis).

### PROMIS Global Short Form (GSF)

Health related quality of life (HRQoL) was assessed using the PROMIS GSF (v1.2) [26]. The instrument consists of 10-items each representing a different domain of health: general health (*Global01*); quality of life (*Global02*); physical health (*Global03*); mental health (*Global04*); social discretionary (*Global05*); physical function (*Global06*); pain (*Global07*); fatigue (*Global08*); social roles (*Global09*); and emotional problems (*Global10*) [20]. Nine of the 10 items are scored on a Likert scale from 1 to 5, with 5 representing best health. *Global08* and *Global10* are reverse coded. Pain was scored from 0 to 10 and was subsequently recoded and reverse scored to a 5-point scale, (worst pain imaginable = 1, no pain = 5). During psychometric tests, Hays and colleagues [20] identified two overall factors, each containing four items: global physical health (GPH) (*Global03, Global6, Global07, Global08*) and global mental health (GMH) (*Global02, Global04, Global05, Global10*). The remaining two items for general health and social roles (*Global01* and *Global09*) were scored as single items separately [20]. The four-item physical and mental health sub-scales have demonstrated an internal consistency reliability of 0.81 and 0.86, respectively [20].

### Procedure

Eligible women attending their first antepartum booking or follow-up visit were approached by either the first author or by their primary midwife and informed of the study aims and requirements. Consenting women provided written consent to participate. Women accessing care by a midwife were provided with a tablet device and completed the baseline survey during the first midwife visit. In a pilot of ten women, time and resource barriers in the antenatal clinic prevented tablet completion for women accessing non-caseload care. These women were sent a survey link by email and text message. Women who failed to respond to the survey within 2 to 3 days were sent a friendly reminder by text message and email. After a second reminder, telephone follow-up was made offering assistance to complete the survey by telephone. All surveys administered at all subsequent time-points were sent to all women using the same text and email protocol. All women were contacted by phone prior to survey administration to gain ongoing consent and discuss survey requirements. Non-responding women were deemed lost to follow up if they failed to complete two consecutive surveys. Surveys were completed during pregnancy: 27-weeks or less (Time 1), 36-weeks (Time 2), birth week (Time 3), 6-weeks postpartum (Time 4) and 26-weeks postpartum (Time 5). This paper reports on HRQoL data obtained at T1, T2, T4 and T5. All participants who commenced the PROMIS GSF at each time point, completed all questions.

### Conceptual framework for instrument evaluation

Standards developed by COSMIN (COnsensus-based Standards for the selection of health Measurement INstruments) were used to guide the psychometric evaluation of the PROMIS GSF. Firstly, standardized terminology and definitions of measurement properties are reported [27]. Next, standards aimed at evaluating the methodological quality of studies on measurement properties [28] are used to guide the psychometric evaluation. Lastly, standards for criteria for good measurement properties are applied to the results to assist interpretation [29].

### Data analysis

Participant sociodemographic characteristics were summarized using descriptive statistics. Frequency and percentages are reported for categorical variables and means and standard deviations or non-parametric alternatives (median and interquartile range) are reported for continuous variables. Normality assumptions were assessed visually, and the Kolmogorov-Smirnov with Lilliefors Significant Correction test value considered. To establish representativeness of the sample National and State perinatal data statistics, compiled by the Australian Institute of Health and Welfare [30–33] and Queensland Health [34] are presented.

### Structural validity and internal consistency reliability

Rasch analysis, using the partial credit model of RUMM2030 [35], evaluated the structural validity of the PROMIS GSF in the sample of women during both pregnancy (baseline) and postpartum period (26-weeks). The total 10-item version of the PROMIS GSF, and the alternate 4-item subscales (global mental health and global physical health) proposed by Hays et al. [20] were evaluated. The procedures adopted were consistent with those recommended by Pallant and Tennant [36] and involve a detailed assessment of the response format, item fit, differential item functioning (item bias), dimensionality of the scale and its targeting. Overall fit of the scale is indicated by a fit residual standard deviation value of 1.4 or less, and a non-significant chi-square statistic. The fit of individual items is assessed, with an individual fit residual value above + 2.5 indicating misfit to the model. The response format is evaluated by inspecting the thresholds and ensuring that none are disordered. The internal consistency reliability of the scale is assessed using a person separation index (PSI) and Cronbach alpha coefficient (α), with values exceeding 0.70 considered acceptable [37], however higher values (e.g. above .85) are desired for tools used for clinical diagnosis. Differential item functioning (DIF), which occurs when groups within the sample respond in a different manner to an individual item, despite equal levels of the underlying characteristic [36] is assessed in RUMM2030 both statistically (using Analysis of Variance) and graphically. DIF was evaluated for age group (18-27 yrs., 28-32 yrs., 33 + years), education level (primary or secondary school versus post-secondary training) and parity (primiparous versus multiparous).

It is important that scales providing a total score are unidimensional, and this is assessed in RUMM2030 using a two-step process. Firstly, principal components analysis is conducted on the residual correlation matrix to identify subsets of items with high positive or negative loadings on the first unrotated factor. Rasch derived scores are calculated for each of these subsets separately and are compared for each respondent using a series of t-tests. A scale is considered unidimensional if fewer than 5% of tests are significant, or if the lower bound of the confidence interval is lower than 5%. The residual correlation matrix is also used to assess local dependency among items, indicated by items showing intercorrelations exceeding .2 above the average interitem correlation value.

### Construct validity

Correlations between the two revised GSF subscales derived from Rasch analysis (Mental Health-Pregnancy Postpartum: MH-PP, and Physical Health-Pregnancy Postpartum: PH-PP) were calculated using Spearman correlation coefficients. The strength of the relationship was interpreted using Cohen’s guidelines [38]; (*r* = .10 to .29 for small; *r* = .30 to .49 for medium and *r* = .50 to 1.0 for a large effect).

Independent samples t-tests were conducted to compare mean scores for mental health and physical health on several grouping variables known to influence health [39–41]. These were: age, parity, education, income, country of birth, obesity, history of mental health disorder, gestation, planned pregnancy, smoker in the past 12 months, pre-pregnancy illicit drug use, stress and previous cesarean birth. Regarding mental health, it was hypothesized that there would be no group differences in terms of age, parity, education, country of birth, obesity, gestation, or previous cesarean birth. It was further hypothesized that women who had low income, a history of mental health disorder, were pre-pregnancy drug users, smoked cigarettes in the past 12 months, had experienced stress in the past 12 months, or whose pregnancy was unplanned, would experience significantly worse mental health compared to their group counterparts.

For physical health, it was hypothesized that there would be no group differences in terms of age, parity, country of birth, history of mental health disorder, gestation, planned pregnancy, stress or previous cesarean birth. Based on the key determinants of health [40] it was further hypothesized that women who were obese, smoked cigarettes, reported pre-pregnancy illicit drug use, low income, or had low education attainment, would experience significantly worse physical health compared to their group counterparts. Homogeneity was assessed using Levene’s test for equality of variance. Effect size is presented as Cohen’s *d* and interpreted as: .2 = small effect, .5 medium effect, and .8 = large effect [38].

### Responsiveness

A one-way repeated-measures analysis of variance (ANOVA) assessed change in mental and physical health scores (responsiveness) over four-time periods. Post hoc tests, with pairwise comparisons between pairs of time-points, were performed for statistically significant results. Significance was set at 5% (*p* ≤ .05). Effect size is presented as partial eta squared and interpreted as: .01 = small, .06 = moderate, and .14 = large effect [38]. Physical and mental health trajectories may differ based on baseline symptom presence and severity [42]. While limited evidence exists on the trajectory of physical health during the peripartum period, poorer physical health is associated with poorer mental health [43]. Pregnancy is associated with poorer physical health in terms of back and pelvic pain, fatigue [42] and incontinence [44] we therefore expected to see a corresponding deterioration in mental health during pregnancy with subsequent improvement by 26-weeks postpartum.

## Results

### Sample characteristics

The socio-demographic characteristics of the sample (*N* = 309) are presented in Table 1. At recruitment most participants were in their second trimester of pregnancy (mean = 19.7 weeks, SD = 3.7, range = 10–27 weeks). Women’s ages ranged from 19 to 43 years with a mean age of 29.8 years (SD = 5.0). Most women were either married or in a de facto relationship (94%). The sample were well educated, with almost 90% of women (*n* = 275) having completed high school (Year 12), a Diploma or Degree. Almost three quarters of women were in paid work (*n* = 227) and one in ten were studying (*n* = 30). Seventy three percent of women were born in Australia. Of those born outside of Australia, the most common places of birth were New Zealand (*n* = 30), United Kingdom (*n* = 16) and Asia (*n* = 14). Less than 10% of women came from the Americas (*n* = 8), Brazil (*n* = 6), South Africa (*n* = 5), Europe (*n* = 4) or Tahiti (*n* = 1). Similarly, one in ten women spoke a language other than English at home (*n* = 31). Of the 90% of women who responded, over half were in the medium highest and highest income groups. While the cohort was similar to National and State populations in terms of age, parity, body mass index (BMI) and country of birth, participants were more likely to be in a relationship.

**Table 1:** Participant characteristics at baseline

Characteristic	Total sample^f^ *n* = 309		State maternity population 2016 *n* = 61,858^a^		National maternity population 2016 *n* = 310,247^a^	
	n	%	n	%	n	%
Gestation (M, SD, Range)	19.7 (3.7)	(10–27)	–	–	–	–
*Age (M, SD, Range)*	29.8 (5.0)	(19–43)	–	–	30.5	–
Less than 20	3	1.0	2110	3.4^a^	7395	2.4 ^a^
20–34	250	80.9	47,559	76.8^a^	232,034	74. ^a^
35 or more	56	18.1	12,189	19.7^a^	70,776	22.8 ^a^
*Parity*						
Primiparous	125	40.5	25,336	41.0^a^	132,842	42.8 ^a^
Multiparous	184	59.5	36,522	59.0^a^	176,671	56.9 ^a^
*Marital status*						
Married/defacto	290	93.9	49,686	80.3^b^	–	–
Single	17	5.5	11,110	18.0 ^b^	–	–
Separated, divorced, widowed	2	0.6	989	1.7 ^b^	–	–
*E ducation*						
Secondary < year 12	34	11.0	–	–	–	–
Secondary to year 12	64	20.7	–	–	–	–
Apprentice / Diploma	100	32.4	–	–	–	–
Tertiary study	111	35.9	–	–	–	–
*Employment/study*						
No paid work or study	72	23.3	–	–	–	–
Paid work	207	67.0	–	–	–	–
Study	10	3.2	–	–	–	–
Both paid work & study	20	6.5	–	–	–	–
*Income $ (Weekly combined)*						
Lowest income (Neg – 999)	45	16.1	544,127	32.0 ^c^	2,661,359	31.5 ^c^
Medium lowest (1000–1499)	74	26.5	271,663	16.0 ^c^	1,278,704	15.2 ^c^
Medium highest (1500–2499)	113	40.5	378,689	22.2 ^c^	1,840,443	21.8 ^c^
Highest income (2500- ≥ 5000)	47	16.8	326,440	19.2 ^c^	1,772,945	21.0 ^c^
*Birth country*						
Australia	224	72.5	45,059	72.8^a^	201,984	65.1^a^
Elsewhere	85	27.5	16,737	27.1 ^a^	106,572	34.4 ^a^
*Body mass index (M, SD, Range)*	24.2 (6.8)	17.2–47.0				
Underweight < 18.5	5	1.6	3431	5.5^a^	12,212	3.9 ^a^
Normal weight 18.5–24.9	141	45.6	31,131	50.3^a^	152,022	49.0 ^a^
Overweight 25.0–29.9	76	24.6	14,228	23.0^a^	76,019	24.5 ^a^
Obese class 1/2 30.0–39.9	55	17.8	10,166	16.4^a^	49,593	16.0 ^a^
Obese class 3 ≥ 40.0	7	2.3	1889	3.0^a^	8762	2.9 ^a^
*Unplanned pregnancy*	88	29.1	–	–	–	–
*Stress in the last 12 months*	82	26.5	–	–	–	–
*Smoking*						
Smoked during pregnancy	34	11.0	–	–	–	9.9 ^a^
Previous smoker, < 12 mth ago	19	6.1	–	–	–	–
Previous smoker, > 12 mth ago	64	20.7	–	–	–	–
*Pre-pregnancy drug use*	33	10.7	–	–	–	–
*Past history of:*						
Diabetes	2	0.6	–	–	–	0.7 ^d^
Hypertension	4	1.3	–	–	–	0.8 ^a^
Mental health disorder	44	14.2	–	–	–	–
Caesarean birth	39	12.6	–	–	–	41.5 ^a^
*Current twin pregnancy*	6	1.9	–	–	–	1.4^e^

Note national and state percentages may not compute to 100% due to missing values

^a^AIHW (2018) [32]

^b^Queensland Health (2016) [34]

^c^Profile Id (2016) [31]

^d^AIHW (2019) [30]

^e^AIHW (2018) [33]

^f^n and % values for women who responded

### Rasch analysis of the PROMIS mental and physical health

#### Mental health subscale

Rasch analysis of the original four items of the Mental Health subscale: items 2 (*quality of life*), item 4 (*mental health*), item 5 (*social discretionary*), and item 10 (*emotional problems*) administered in pregnancy at baseline indicated some degree of misfit among items (see Table 2: Analysis 1) with a fit residual standard deviation value of 2.35, exceeding the recommended value of 1.4. Item 10 (*emotional problems*) recorded an individual Fit Residual value of 2.75, suggesting misfit to the Rasch model. Removal of this item improved overall fit and resulted in a 3-item scale (see Table 2: Analysis 2) with no evidence of misfit items, no differential item functioning by age, education or parity, and no evidence of local dependency. The scale met the requirements for unidimensionality, with the lower bound of the confidence interval around the percentage of cases with significantly different subtest scores (2.8%) falling below the 5% criteria (see Table 2: Analysis 2). The internal consistency reliability was adequate, with a PSI value of .77 and Cronbach’s alpha value of .84.

**Table 2:** Summary of results of Rasch analysis: original and revised subscales during pregnancy and postpartum

Subscale	Analysis	Item No.	Overall model fit	Item fit residual mean (SD)	Person fit residual mean (SD)	PSI	Cronbach alpha	% sig t-tests^a^
Pregnancy (Baseline)								
Original Mental health (4 items)	1	2, 4, 5, 10	Chi sq. = 14.21 df = 16, *p* = .58	.12 (2.35)	−.44 (1.22)	.79	.83	5.24% (CI: 2.8–7.8)
Revised Mental Health (remove item 10)	2	2, 4, 5	Chi sq. = 11.79, df = 9, *p* = .22	−.01 (1.12)	−.52 (1.01)	.77	.84	3.65%
Revised Mental Health (add item 9)	3	2, 4, 5, 9	Chi sq. = 8.45, df = 16, *p* = .93	−.21 (.82)	−.63 (1.27)	.84	.88	5.4% (CI: 2.8–8.0)
Original Physical Health (4 items)	4	3, 6, 7, 8	Chi sq. = 19.03, df = 16, *p* = .27	−.09 (1.06)	−.37 (.99)	.64	.69	2.63%
Revised Physical Health (add item 1)	5	1, 3, 6, 7, 8	Chi sq. = 29.14, df = 20, *p* = .08	−.12 (1.45)	−.39 (1.09)	.74	.76	3.62%
Postpartum (26-weeks)								
Revised Mental Health	6	2, 4, 5, 9	Chi sq. = 13.86, df = 12, *p* = .31	−.18 (1.0)	−.48 (1.01)	.84	.87	3.65%
Revised Physical Health	7	1, 3, 6, 7, 8	Chi sq. = 34.82, df = 15, *p* = 0.02	−.62 (2.08)	−.42 (.88)	.72	.75	5.6% (CI: 2.8–8.4)

*Chi sq* chi-square, *df* degrees of freedom, *p* probability, *PSI* person separation index, *CI* confidence interval

^a^Confidence interval only reported if the % value exceeds 5%

An additional Rasch analysis assessed the suitability of including item 9 (*social roles*) in the revised 3-item Mental Health subscale. This alternative subscale showed good fit to the Rasch model (see Table 2: Analysis 3). This 4-item solution showed a substantial improvement in the internal consistency reliability, with an increase in the PSI value to .84, and Cronbach’s α value to .88.

**Fig. 2:**
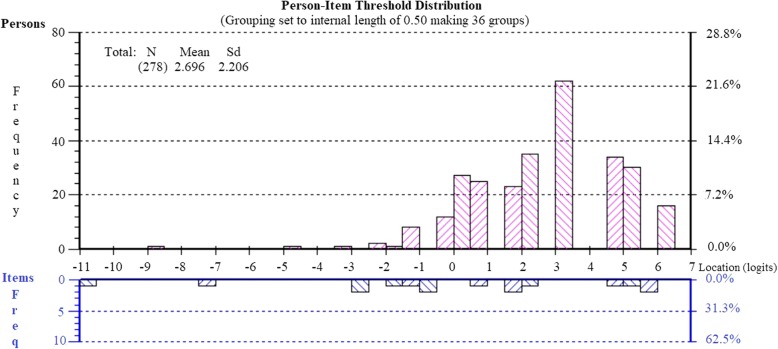
Person-item threshold distribution for the revised mental health subscale

To assess suitability of the revised four-item version of the Mental Health subscale (items 2, 4, 5, 9) for use with postpartum women, Rasch analysis was conducted on the responses obtained from respondents at 26-weeks post birth. The subscale showed good fit to the Rasch model, with no misfitting items, no differential item functioning for age, education or parity, and no evidence of local dependency or multidimensionality (see Table 2: Analysis 6). The internal consistency reliability of the subscale was good (PSI = .84). The Person-Item Threshold Distribution map is shown in Fig. 2, suggesting appropriate targeting of the items to this sample of women, with no evidence of floor or ceiling effects.

#### Physical health subscale

**Fig. 3:**
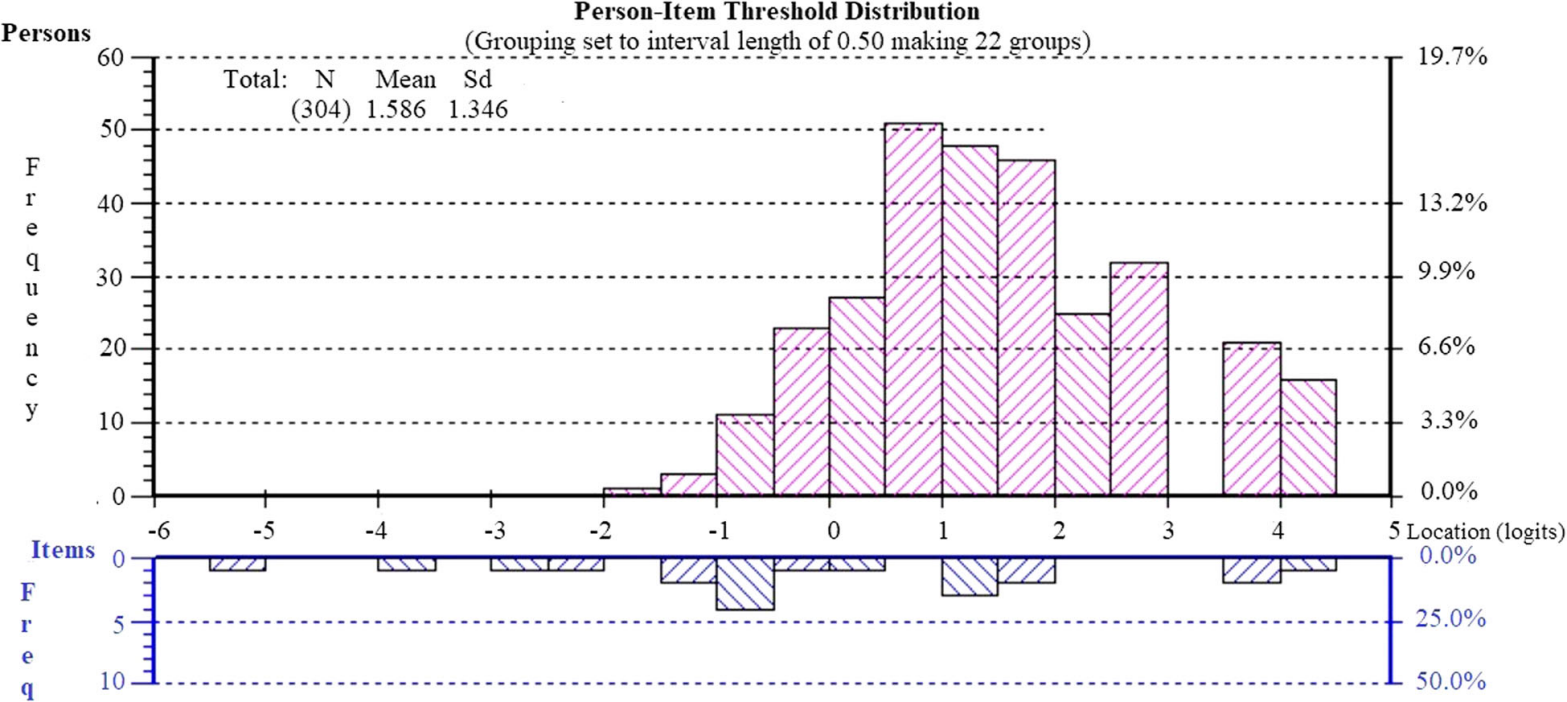
Person-item threshold distribution for the revised physical health subscale

Rasch analysis of the original four items of the Physical Health subscale: item 3 (*physical health*), item 6 (*physical function*), item 7 (*pain*) and item 8 (*fatigue*) administered to pregnant women at baseline showed adequate fit to the Rasch model (see Table 2: Analysis 4), however the internal consistency was quite low (PSI = .64, Cronbach α = .69). Assessment of an alternative version of the Physical Health subscale which included item 1 (*general health*), showed good fit to the model, with a substantial improvement in the PSI value (from .64 to .74) and Cronbach’s α value (from .69 to .76). The final 5-item version of the Physical Health subscale showed good fit to the Rasch model, no evidence of misfitting items, no differential item functioning for age, education or parity and no local dependency (Table 2: Analysis 5). The subscale showed no evidence of multidimensionality, with the percentage of persons with significantly different subtest scores not exceeding the criteria of 5% (see Table 2, Analysis 5). The Person-Item Threshold Distribution map, presented in Fig. 3, also supports the appropriate targeting of the level of physical health in this cohort of pregnant women. No floor or ceiling effects were detected.

Rasch analysis of the revised 5-item version of the Physical Health subscale was also conducted on data obtained at 26-weeks post birth. There were no misfitting items, no differential functioning for age, education or parity, and no local dependency. Analyses supported its unidimensionality (see Table 2, Analysis 7).

Based on these results, two revised subscales are proposed for use during pregnancy and postpartum period (see Table 3). The proposed physical health subscale (PH-PP) includes five items: item 1 (*general health*), item 3 (*physical health*), item 6 (*physical function*), item 7 (*pain*), and item 8 (*fatigue*). The proposed mental health subscale (MH-PP) includes four items: item 2 (*quality of life*), item 4 (*mental health*), item 5 (*social discretionary*), and item 9 (*social roles*). Total scores for these scales were calculated by summing item scores (on a 1–5 range) and dividing by the number of items in the scale. Higher scores indicated better physical and mental health.

**Table 3:** Proposed revised measure of global health in pregnancy and postpartum: five-item physical health and four-item mental health subscales

Item	Domain	Item wording	Item response (score)				
Physical Health Subscale							
Item 1	General health	In general, would you say your health is: ......	Excellent (5)	Very good (4)	Good (3)	Fair (2)	Poor (1)
Item 3	Physical health	In general, how would you rate your physical health?	Excellent (5)	Very good (4)	Good (3)	Fair (2)	Poor (1)
Item 6	Physical function	To what extent are you able to carry out your everyday physical activities such as walking, climbing stairs, carrying groceries, or moving a chair?	Completely (5)	Mostly (4)	Moderately (3)	A little (2)	Not at all (1)
Item 7	Pain^a^	In the past 7 days, how would you rate your pain on average?	No pain (5)	Score 1–3 (4)	Score 4–6 (3)	Score 7–9 (2)	Worst pain (1)
Item 8	Fatigue	In the past 7 days, how would you rate your fatigue on average?	None (5)	Mild (4)	Moderate (3)	Severe (2)	Very severe (1)
Mental Health Subscale							
Item 2	Quality of life	In general, would you say your quality of life is:	Excellent (5)	Very good (4)	Good (3)	Fair (2)	Poor (1)
Item 4	Mental health	In general, how would you rate your mental health, including your mood and your ability to think?	Excellent (5)	Very good (4)	Good (3)	Fair (2)	Poor (1)
Item 5	Social discretionary	In general, how would you rate your satisfaction with your social activities and relationships?	Excellent (5)	Very good (4)	Good (3)	Fair (2)	Poor (1)
Item 9	Social roles	In general, please rate how well you carry out your usual social activities and roles. (This includes activities at home, at work and in your community, and responsibilities as a parent, child, spouse, employee, friend, etc.)	Excellent (5)	Very good (4)	Good (3)	Fair (2)	Poor (1)

Source: PROMIS Health Organization and PROMIS Cooperative Group [26]

^a^Pain scored from 0 to 10: 0 = No pain, 10 = Worst pain imaginable and subsequently reverse scored and recoded to 5 responses

Spearman correlation coefficients showed a strong correlation between the MH-PP and PH-PP subscales during pregnancy at baseline (*rho *= .64) and at 26-weeks postpartum (*rho* = .67), indicating that they shared 41.0 and 44.9% of variance respectively.

#### Trajectory of mental and physical health during pregnancy and postpartum

**Fig. 4:**
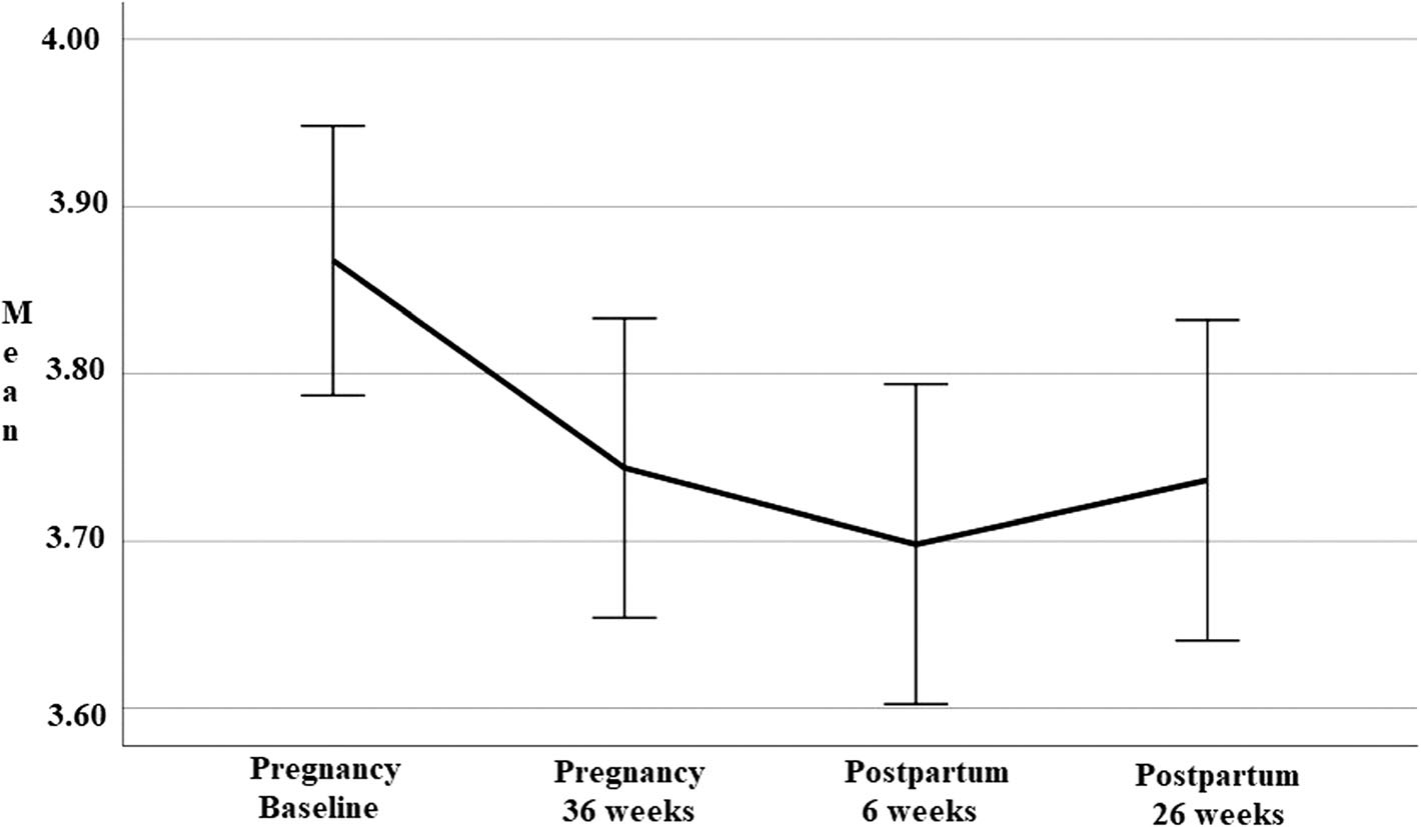
Mental health (MH-PP) scores at four time-points over pregnancy and postpartum

**Fig. 5:**
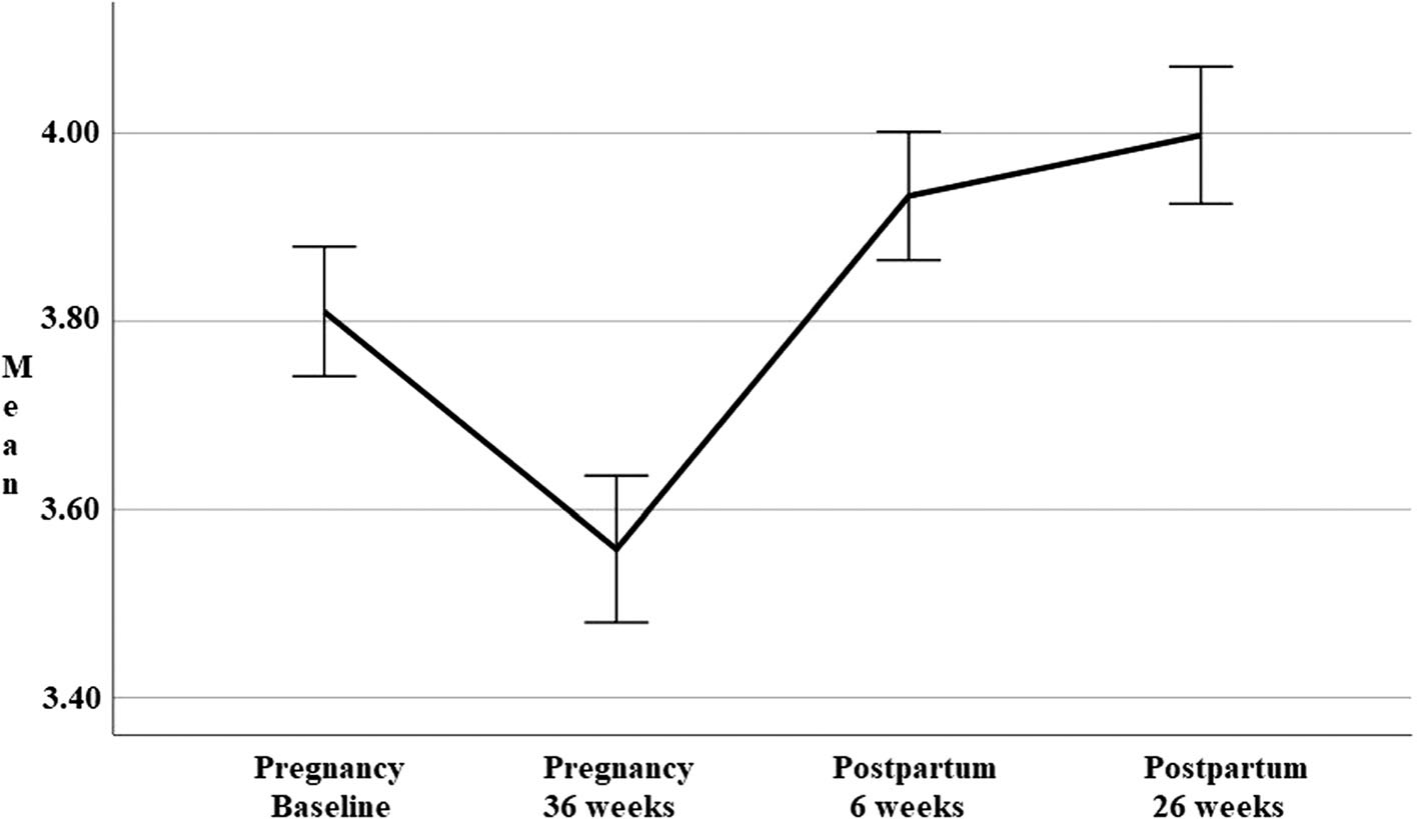
Physical health (PH-PP) scores at four time-points over pregnancy and postpartum

Descriptive statistics for the revised mental health (MH-PP) and physical health (PH-PP) subscales obtained over four time-points during pregnancy and postpartum are presented in Table 4. A series of one-way repeated measures ANOVAs were conducted to compare scores on the revised Mental Health (MH-PP) and Physical Health subscale (PH-PP) during pregnancy (baseline and 36-weeks) and postpartum period (6- and 26-weeks). Line-charts presented in Figs. 4 and 5 show the trajectory of physical and mental health over the course of pregnancy and postpartum.

**Table 4:** Revised mental health (MH-PP) and physical health (PH-PP) scores over 4 time-points

	*n*	Mean (SD)	Median (IQR)	Range
*Mental health* ^*a*^ *(MH-PP)*				
Pregnancy Baseline	308	3.87 (0.72)	4.00 (1.00)	1.25–5
Pregnancy 36 weeks	276	3.74 (0.76)	3.75 (1.00)	1.25–5
Postpartum 6 weeks	260	3.70 (0.78)	3.75 (1.19)	1.25–5
Postpartum 26 weeks	238	3.74 (0.75)	3.75 (1.00)	1.50–5
*Physical health* ^*b*^ *(PH-PP)*				
Pregnancy Baseline	308	3.81 (0.61)	3.80 (0.80)	2.00–5
Pregnancy 36 weeks	276	3.56 (0.66)	3.60 (1.15)	1.20–5
Postpartum 6 weeks	260	3.93 (0.56)	4.00 (0.80)	1.60–5
Postpartum 26 weeks	238	4.00 (0.57)	4.00 (0.80)	2.00–5

^a^MH-PP subscale calculated by summing items 2, 4, 5, 9 and dividing by 4

^b^PH-PP subscale calculated by summing items 1, 3, 6, 7, 8 and dividing by 5

Figure 4 shows mental health scores on the MH-PP were highest at baseline, reduced during pregnancy (36-weeks) and early postpartum (6-weeks) before returning to values seen in late pregnancy at 26-weeks postpartum, suggesting a deterioration of mental health from that seen at baseline at all time points. The effect was significant for time (Wilks’ Lambda = .94, *F* (3, 218) = 5.03, *p* = .002). The partial eta squared value .07 indicates a moderate effect size. Four paired samples t-tests were used to make post hoc comparisons between time points. There was a significant difference between MH-PP scores from baseline to 36-weeks (*p* = .03), baseline to early postpartum (*p* = .003) and baseline to 26-weeks postpartum (*p* = .03). There were no statistically significant differences between any other time points.

Figure 5 shows physical health scores (PH-PP) deteriorated during pregnancy (to 36-weeks), before steadily improving to values exceeding baseline scores at both postpartum time points (6- and 26-weeks). Highest scores were seen at 26-weeks postpartum. This change in PH-PP scores demonstrated a significant effect for time (Wilks’ Lambda = .69, *F* (3, 218) = 32.28, *p* < .001). The partial eta squared value .31 indicated a very large effect size. There were significant differences in scores from late pregnancy (36-weeks) to all other time points (*p* < .001). While postpartum scores were significantly different to pregnancy scores, there were no difference demonstrated postpartum from 6- to 26-weeks.

#### Construct validity of the revised mental health subscale

A series of independent sample t-tests compared MH-PP scores obtained during pregnancy (baseline) and postpartum (26-weeks) for several groups (see Table 5). Women who reported low income, unplanned pregnancy, pre-pregnancy drug use or stress in the previous 12 months had significantly lower mental health scores during pregnancy. While the effect was large or very large during pregnancy, no difference was seen in the postpartum period. For women who reported smoking in the previous 12 months or a history of mental health disorder, mental health scores were lower than their group counterparts, both during pregnancy and postpartum period, with very large effect sizes. The largest effect was for women with a history of mental health disorder during pregnancy. No significant group differences were seen in terms of age, parity, education, country of birth, obesity, gestation at baseline, or previous cesarean birth.

**Table 5:** Group differences on mental health (MH-PP) scores during pregnancy (baseline) and postpartum (26-weeks)

Characteristic	Groups	n	M (SD)	Mean Difference (95% CI)	t	df	*p*	Cohen’s *d*
Age								
*Pregnancy*				0.09 (− 0.30–0.11)	0.89	306	.37	0.12
	≥ 35	56	3.79 (0.78)					
	< 35	252	3.88 (0.71)					
*Postpartum*				0.11 (− 0.35–0.13)	0.90	236	.37	0.14
	≥ 35	46	3.65 (0.78)					
	< 35	192	3.76 (0.75)					
Parity								
*Pregnancy*				0.12 (0.08 – − 0.28)	1.42	306	.16	0.17
	Nulliparous	125	3.94 (0.78)					
	Multiparous	183	3.82 (0.67)					
*Postpartum*				0.08 (− 0.12–0.27)	0.76	236	.45	0.09
	Nulliparous	100	3.78 (0.81)					
	Multiparous	138	3.71 (0.71)					
Education^a^								
*Pregnancy*				0.14 (−0.31–0.03)	1.61	306	.12	0.20
	Low	97	3.77 (0.69)					
	High	211	3.91 (0.73)					
*Postpartum*				0.03 (−0.24–0.18)	0.29	236	.77	0.04
	Low	71	3.72 (0.74)					
	High	167	3.75 (0.76)					
Income^b^								
*Pregnancy*				0.37 (0.21–0.54)	4.36	276	**<.001**	**0.53**
	High	160	4.02 (0.68)					
	Low	118	3.64 (0.74)					
*Postpartum*				0.15 (−0.05–0.36)	1.47	214	.14	0.21
	High	129	3.78 (0.74)					
	Low	87	3.62 (0.76)					
Country of birth								
*Pregnancy*				0.11 (−0.07–0.29)	1.20	306	.23	0.15
	Australia	223	3.90 (0.72)					
	Other	85	3.79 (0.71)					
*Postpartum*				0.10 (−0.12–0.31)	0.81	236	.42	0.12
	Australia	175	3.76 (0.76)					
	Other	63	3.67 (0.72)					
Obesity^c^								
*Pregnancy*				0.11 (−0.31–0.09)	1.11	281	.27	0.15
	Obese	62	3.78 (0.80)					
	Non-obese	221	3.89 (0.67)					
*Postpartum*				0.09 (−0.33–0.15)	0.74	220	.46	0.10
	Obese	51	3.66 (0.78)					
	Non-obese	171	3.74 (0.76)					
Mental health^d^								
*Pregnancy*^e^				0.47 (0.20–0.75)	3.44	50.61^e^	**.001**	**0.61**
	Yes	43	3.46 (0.86)					
	No	265	3.93 (0.67)					
*Postpartum*				0.29 (0.02–0.57)	2.12	236	**.04**	**0.36**
	Yes	34	3.49 (0.88)					
	No	204	3.78 (0.72)					
Gestation								
*Pregnancy*				0.08 (−0.14–0.29)	0.71	306	.48	0.11
	≤ 15 weeks	52	3.80 (0.76)					
	> 15 weeks	256	3.88 (0.71)					
*Postpartum*				0.07 (−0.19–0.33)	0.52	236	.60	0.09
	≤ 15 weeks	38	3.68 (0.87)					
	> 15 weeks	200	3.75 (0.73)					
Planned pregnancy								
*Pregnancy*^e^				0.22 (0.03–0.42)	2.30	138.98^e^	**.02**	**0.30**
	Planned	214	3.93 (0.68)					
	Unplanned	87	3.71 (0.80)					
*Postpartum*				0.13 (−0.08–0.45)	1.22	235	.22	0.17
	Planned	170	3.78 (0.74)					
	Unplanned	67	3.65 (0.77)					
Smoker in last 12 months								
*Pregnancy*^e^				0.24 (0.46–0.02)	2.21	64.39^e^	**.03**	**0.36**
	Smoker	52	3.61 (0.74)					
	Non-smoker	256	3.85 (0.58)					
*Postpartum*				0.23 (0.43–0.03)	2.25	236	**.03**	**0.36**
	Smoker	37	3.81 (0.66)					
	Non-smoker	201	4.03 (0.54)					
Pre-pregnancy drug use								
*Pregnancy*				0.31 (0.05–0.58)	2.36	306	**.02**	**0.41**
	Drug use	32	3.59 (0.82)					
	No drug use	276	3.90 (0.70)					
*Postpartum*				0.15 (−0.48–0.19)	0.85	236	.40	0.22
	Drug use	21	3.87 (0.61)					
	No drug use	217	3.72 (0.76)					
Stress in the last 12 months								
*Pregnancy*				0.26 (0.08–0.44)	2.85	296	**.01**	**0.36**
	Stress	81	3.69 (0.76)					
	No stress	217	3.95 (0.69)					
*Postpartum*				0.08 (−0.13–0.30)	0.75	233	.45	0.11
	Stress	65	3.69 (0.70)					
	No stress	170	3.77 (0.77)					
Previous caesarean								
*Pregnancy*				0.11 (−0.14–0.35)	0.86	306	.39	0.14
	Yes	39	3.78 (0.66)					
	No	269	3.88 (0.73)					
*Postpartum*				0.13 (−0.16–0.43)	0.88	236	.38	0.18
	Yes	29	3.62 (0.67)					
	No	209	3.75 (0.76)					

Bolded *p and* Cohen’s *d* indicates significant results

^a^Low education = Secondary school year 12 or less; High education = College apprenticeship/Diploma or University

^b^Low income = Nil - $1499, High income = $1500 or more;

^c^Obese = BMI ≥ 30, Non obese = BMI < 30

^d^History of mental health disorder

^e^Violation of assumption of equal variances

#### Construct validity of the revised physical health subscale

A series of independent sample t-tests compared PH-PP scores obtained during pregnancy (baseline) and postpartum period (26-weeks) for several groups (see Table 6). Women who smoked, were on low income, were obese or who had a history of mental health disorder reported poorer physical health scores during both pregnancy and postpartum period. Women with low education attainment reported poorer physical health in pregnancy, but not during the postpartum period. Women who reported pre-pregnancy drug use had poorer antepartum physical health, but this was not seen in the postpartum period. Those women who experienced unplanned pregnancy or stress in the last 12 months reported poorer physical health, but only during pregnancy. Effect sizes for these comparisons were largest for the impact of low income in the postpartum period (Cohen’s *d* = 0.51) and history of mental health disorder during pregnancy (Cohen’s *d* = 0.49). No significant group differences in physical health were seen in terms of age, country of birth, gestation at baseline, and previous cesarean birth.

**Table 6:** Group differences on physical health (PH-PP) scores during pregnancy (baseline) and postpartum (26-weeks)

Characteristic	Groups	n	M (SD)	Mean Difference (95% CI)	t	df	*p*	Cohen’s *d*
Age								
*Pregnancy*				0.11 (−0.07–0.29)	1.21	306	.23	0.17
	≥ 35	56	3.90 (0.69)					
	< 35	252	3.79 (0.59)					
*Postpartum*				0.15 (−0.34–0.02)	1.70	236	.09	0.27
	≥ 35	46	3.87 (0.63)					
	< 35	192	4.03 (0.55)					
Parity								
*Pregnancy*				0.15 (0.01–0.23)	2.11	306	**.04**	**0.24**
	Nulliparous	125	3.90 (0.62)					
	Multiparous	183	3.75 (0.61)					
*Postpartum*				0.04 (−0.11–0.19)	0.57	236	.57	0.07
	Nulliparous	100	4.02 (0.60)					
	Multiparous	138	3.98 (0.55)					
Gestation								
*Pregnancy*				0.01 (−0.19–0.12)	0.06	306	.95	0.02
	≤ 15 weeks	52	3.82 (0.64)					
	> 15 weeks	256	3.81 (0.61)					
*Postpartum*				0.04 (−0.15–0.23)	0.41	236	.67	0.07
	≤ 15 weeks	38	3.96 (0.65)					
	> 15 weeks	200	4.00 (0.55)					
Education^a^								
*Pregnancy*				0.15 (0.30–0.01)	2.05	306	**.04**	**0.24**
	Low	97	3.71 (0.62)					
	High	211	3.86 (0.61)					
*Postpartum*				0.13 (−0.29–0.03)	1.60	236	.11	0.23
	Low	71	3.91 (0.56)					
	High	167	4.04 (0.57)					
Income^b^								
*Pregnancy*				0.23 (0.08–0.37)	3.09	276	**.002**	**0.36**
	High	160	3.92 (0.60)					
	Low	118	3.70 (0.61)					
*Postpartum*				0.23 (0.14–0.44)	3.73	214	. < .**001**	**0.51**
	High	129	4.10 (0.52)					
	Low	87	3.81 (0.61)					
Country of birth								
*Pregnancy*				0.03 (−0.12–0.18)	0.39	306	.70	0.04
	Australia	223	3.82 (0.60)					
	Other	85	3.79 (0.64)					
*Postpartum*				0.05 (−0.11–0.21)	0.58	236	.56	0.08
	Australia	175	4.01 (0.54)					
	Other	63	3.96 (0.65)					
Obesity^c^								
*Pregnancy*				0.21 (0.38–0.04)	2.43	281	**.02**	**0.34**
	Obese	62	3.63 (0.66)					
	Non obese	221	3.84 (0.59)					
*Postpartum*				0.23 (0.41–0.05)	2.5	220	**.01**	**0.40**
	Obese	51	3.80 (0.59)					
	Non obese	171	4.03 (0.56)					
Mental health^d^								
*Pregnancy*^e^				0.33 (0.08–0.57)	2.71	50.28^e^	**.01**	**0.49**
	Yes	43	3.53 (0.75)					
	No	265	3.86 (0.58)					
*Postpartum*				0.22 (0.01–0.42)	2.07	236	**.04**	**0.37**
	Yes	34	3.81 (0.64)					
	No	204	4.03 (0.55)					
Smoker in last 12 months								
*Pregnancy*^e^				0.24 (0.46–0.02)	2.21	64.39^e^	**.01**	**0.36**
	Smoker	52	3.61 (0.74)					
	Non-smoker	256	3.85 (0.58)					
*Postpartum*				0.23 (0.43–0.03)	2.25	236	**.03**	**0.36**
	Smoker	37	3.81 (0.66)					
	Non-smoker	201	4.03 (0.54)					
Pre-pregnancy drug use								
*Pregnancy*				0.37 (0.60–0.15)	3.32	306	**.001**	0.60
	Drug use	32	3.48 (0.63)					
	No drug use	276	3.85 (0.60)					
*Postpartum*				0.03 (−.22–0.29)	0.22	236	.83	0.05
	Drug use	21	3.97 (0.54)					
	No drug use	217	4.00 (0.57)					
Planned pregnancy								
*Pregnancy*				0.17 (0.02–0.32)	2.21	229	**.03**	**0.27**
	Planned	214	3.87 (0.59)					
	Unplanned	87	3.70 (0.65)					
*Postpartum*				0.09 (−0.06–0.25)	1.15	235	.25	0.18
	Planned	170	4.03 (0.58)					
	Unplanned	67	3.93 (0.54)					
Stress in the last 12 months								
*Pregnancy*				0.19 (0.04–0.35)	2.43	296	**.02**	**0.31**
	Stress	81	3.68 (0.63)					
	No stress	217	3.87 (0.60)					
*Postpartum*				0.08 (−0.08–0.24)	0.94	233	.35	0.14
	Stress	65	3.95 (0.56)					
	No stress	170	4.03 (0.56)					
Previous caesarean								
*Pregnancy*				0.07 (−0.13–0.28)	0.67	306	.50	0.12
	Yes	39	3.75 (0.51)					
	No	269	3.82 (0.63)					
*Postpartum*				0.03 (−0.19–0.25)	0.25	236	.80	0.05
	Yes	29	3.97 (0.62)					
	No	209	4.00 (0.56)					

Bolded *p and* Cohen’s *d* indicates significant results

^a^Low education = Secondary school year 12 or less; High education = College apprenticeship/Diploma or University

^b^Low income = Nil - $1499, High income = $1500 or more;

^c^Obese = BMI ≥ 30, Non obese = BMI < 30

^d^History of mental health disorder

^e^Violation of assumption of equal variances

## Discussion

This study addressed calls from the International Consortium of Health Outcomes Measurement’s (ICHOM) working party [19] to validate and refine the instruments included in their Standard Set of Outcome Measures for Pregnancy and Childbirth. Rasch analysis of the PROMIS Mental Health and Physical Health subscales assessed the suitability of subscale items and response format, and to detect any potential item bias, local dependency and multi-dimensionality and targeting. For the Mental Health subscale an optimal solution, showing good psychometric properties, was obtained by removing item 10 (*emotional problems*) and adding item 9 (*social roles*). This revised four-item subscale (MH-PP) recorded good internal consistency reliability, with no evidence of problems with the response format, item bias, local dependency, or multi-dimensionality. The original 4-item version of the Physical Health subscale was found to have poor internal consistency reliability, which was improved by the addition of item 1 (*general health*) to form a revised 5-item version of the scale (PH-PP) with adequate psychometric properties. The revised MH-PP was found to be sensitive to differences in groups according to history of mental health, income, smoking and drug use, stress levels and planned versus unplanned pregnancy. Differences in scores on the revised PH-PP were detected for groups based on obesity, income, pre-pregnancy drug use, smoking status, stress, and history of mental health disorders. Scores on both subscales recorded significant changes across the four time-points, spanning pregnancy and postpartum.

### Building on the contribution of others

This study extended research on the psychometric properties of the PROMIS by using modern test theory [22] such as Rasch analysis, to evaluate the properties of the scale in a sample of women, during both pregnancy and postpartum period. This differed from previous studies, such as Hays et al. [20], who used classical test theory approaches (exploratory and confirmatory factor analysis) and samples containing a diverse range of participants. Classic test theory analyses raw scores to test assumptions underlying a given measure. Items are thus summed without weighting or standardization to produce a score [45]. Rasch methodology on the other hand, is a sophisticated and robust method based on a mathematical item response model that affords several advantages over the classic test theory. The main difference being in the management and analysis of data [21]. In Rasch modelling, the probability of a specified response is modelled as a logistic function of the difference between the person and item parameter. Item locations are subsequently scaled (item calibration) and person locations are measured on the same scale. Each item and person estimate has an associated standard error of measurement. Rasch methodology thus enables the transformation of ordinal summed scores into linear measurements. Rasch methodology prioritizes the Rasch model and revisits hypotheses in the event of ill-fitting data [22]. Rasch methodology moves beyond traditional psychometric methods to rigorously evaluate the PROMIS GSF. Further, our study findings build on those of Lundsberg et al. [25] who investigated the use of PROMIS in a sample of women in their first trimester (M = 9 weeks, SD = 4.6), seeking pregnancy testing or services for termination of pregnancy in antepartum clinics in New Haven, USA. The sample was culturally diverse. Almost three-quarters of all pregnancies were unplanned (72.7%). Almost 40% of participants planned termination, adoption, or were unsure of plans and therefore likely to be experiencing considerable stress.

### Scale modifications for maternity populations

In the current study, modifications to the original PROMIS subscales, and the alternative subscale structure recommended by Hays, et al. [20], were required to ensure they were appropriate for use with women during pregnancy and postpartum period. It was necessary to remove item 10 (*emotional problems*) from the Mental Health subscale and add item 9 (*satisfaction with social roles).* The four-item version of the Physical Health subscale (items 3, 6, 7, 8) proposed by Hays et al. recorded relatively poor internal consistency reliability (α = .69). Adding an additional item from the original PROMIS (item 1: *general health*) improved the Cronbach alpha levels in both pregnancy and postpartum period to acceptable levels (referred to as the Physical Health-Pregnancy and Postpartum: PH-PP). The scales appropriately assessed levels of mental and physical health in the current sample of women, and both distributions approximated a normal distribution, with no evidence of floor or ceiling effects.

### Findings related to reliability and validity

Preliminary assessment of the construct validity of the MH-PP and PH-PP revealed significant differences in MH-PP scores for history of mental health disorder, stress, income, smoking, drug use and planned pregnancy. The PH-PP successfully distinguished groups based on health-related factors such as obesity, income, smoking and drug use, mental health disorder, stress levels and education attainment. Differences were also detected for parity, and planned pregnancy. These findings provide preliminary support for the construct validity of the revised PROMIS scales, for use in pregnancy and postpartum period.

Further support for the revised subscales comes following the application of criteria for good measurement properties, as outlined by Prinsen and colleagues [29]. Structural validity using Rasch analysis demonstrated no violations of unidimensionality, local independence or monotonicity. Similarly, with regards to construct validity and responsiveness, at least 75% of results were in accordance with the hypotheses. All reported measurement properties of the two revised subscales, in terms of structural validity, internal consistency, hypothesis testing for construct validity and responsiveness, met the highest rating required of a good measurement property, indicating confidence in the psychometric ability of the revised scales.

### Strengths and weaknesses

This study has two major strengths. Firstly, the comprehensive psychometric evaluation, including Rasch Analysis fully explored all aspects of the PROMIS Global Short Form, informing the development of two revised subscales to measure mental and physical health in a maternity population. Rasch analysis enhanced the findings of Hays et al. [20] and the item calibrations for measuring health related quality of life in terms of mental and physical health were not sample dependent. Further, to address the limitations of heterogeneity in terminology and definitions of measurement properties, consensus-based standards guided the study design and psychometric analysis of the PROMIS GSF. Standards developed by COSMIN were used to guide the psychometric evaluation of the PROMIS GSF and consensus-agreed taxonomy was used [27]. Employing these standards contributes to a transparent and standardized approach to the psychometric evaluation and will support the evidence-based inclusion of the PROMIS GSF within the ICHOM core outcome set.

This study was conducted with 309 women from one birthing facility in Australia. According to the broad aims, sample size was not calculated to measure a specified difference in health-related quality of life. While few studies using Rasch analysis conduct a priori sample size and power determination [46], inadequate sample size can lead to inaccurate results. While several researchers have studied the effect of sample size on power and effect size using simulation and Rasch techniques [47, 48], little consensus exists. The sample size in this study (*n* = 309) exceeded the size recommended by Linacre 1994 [49] to ensure accurate estimation (99%) of person estimates within +/− logits.

Our comparisons with National and State maternity populations showed that the sample was similar to the general maternity population in several ways. However, some group differences were identified. Participants were more likely to be in a relationship and more likely to be in higher income groups compared to Australian National and State averages, outcomes known to positively impact a woman’s health and wellbeing and subsequent health related quality of life [40]. It is possible that participants experienced better physical and mental health outcomes compared to other Australian samples. Replication of this study using larger numbers in diverse maternity populations is recommended.

### Research implications

Our analysis revealed two revised scales to measure mental and physical health in women during pregnancy and postpartum period. Content validity was not evaluated. Findings using the original scales during pregnancy show lower reliability for the physical health subscale compared to that of the mental health subscale (α = .69 vs 0.83) These findings are mirrored by Lundsberg et al. (0.63 and 0.85, respectively) [25]. The content validity specific to a maternity population were not explored in the current study. Future researchers may wish to consider a thorough evaluation of content validity in any future development of a population-specific woman-reported outcome instrument, aimed at measuring HRQoL in maternity populations. The revised physical and mental health scores could be used to evaluate the effect of different models of maternity care provision or other lifestyle interventions on maternal physical and mental health. The evaluation of the impact of maternity care provision and outcomes related to care on women’s HRQoL should be included in future evaluations.

### Clinical implications

This study addresses the call from ICHOM for researchers to validate the instruments included in their Standard Set of Outcome Measures for Pregnancy and Childbirth [19]. Inclusion of this valid and reliable measure will make a positive contribution to the core outcome set and will facilitate comparison and benchmarking of one component of the ICHOM Standard Set. The current climate of contemporary maternity care demands that care meets the needs of women and their babies, yet little is known about the impact of maternity care provision, or pregnancy and birth experiences and outcomes on maternal HRQoL. Results of this study will inform future research into the impact of maternity care provision on HRQoL using valid and reliable tools.

## Conclusion

This comprehensive psychometric analysis, which included Rasch analysis, demonstrated that a revised version of the PROMIS Global Short Form was better able to measure mental and physical health in a pregnant and postpartum population compared to the original generic instrument. While further evaluation of the revised scale is needed on other maternity populations, findings support the clinical and research application of the revised PROMIS GSF within the ICHOM Standard Set for Pregnancy and Childbirth.

## Supplementary information

**Additional file 1.** Details of variables used in analysis.

## Data Availability

The de-identified dataset used and analysed for this study is available from the corresponding author upon reasonable request so that appropriate data transfer agreements can be established.

## Abbreviations

AIHW: Australian Institute of Health and Welfare
ANOVA: Analysis of variance
BMI: Body mass index
CI: Confidence interval
COSMIN: COnsensus-based Standards for the selection of health Measurement INstruments
df: Degrees of freedom
DIF: Differential item functioning
GMH: Global mental health
GPH: Global physical health
GSF: Global Short Form
HRQoL: Health related quality of life
ICHOM: International Consortium for Health Outcomes Measurement
MH-PP: Mental Health-Pregnancy Postpartum
MoMeNT: Models Meeting Needs Over Time
OECD: Organization for Economic Cooperation and Development
PH-PP: Physical Health-Pregnancy Postpartum
PROMIS: Patient Reported Outcomes Measurement Information System
PSI: Person separation index
SD: Standard deviation
US: United States
