# Supplementary material for: Measuring physical and mental health during pregnancy and postpartum in an Australian childbearing population - validation of the PROMIS Global Short Form
Source: BMC Pregnancy Childbirth. 2019 Oct 22;19:370. doi: 10.1186/s12884-019-2546-6 (PMC6805680; doi:10.1186/s12884-019-2546-6)
Supplement: Supplementary file 1 — Additional file 1. Details of variables used in analysis. [file 12884_2019_2546_MOESM1_ESM.docx]

# **Additional File 1: Details of variables used in analysis**

**1.1 What is todays date? _ _ / _ _ / _ _**

**1.2 What is your date of birth? _ _ / _ _ / _ _**

**1.3 How many weeks pregnant are you today? ________weeks**

**1.4 What date is your baby due? _ _ / _ _ / _ _**

# **2.1** What is your *current* marital status?

**(*Please mark one box only*)**

- Single, not living with partner
- Married/De facto
- Divorced/separated
- Widowed

**2.2 What is the highest level of education you have attained? (*Please mark one box only*)**

- Primary school only
- Secondary school less than year 12
- Secondary school year 12
- Completed an apprenticeship or diploma
- Completed a tertiary degree/ postgraduate studies

**2.3 Are you currently involved in paid work or study?**

- No
- Yes, paid work
- Yes, studying
- Both paid work and study

**2.4 In which country were you born?**

**(*Please mark one box only*)**

- Australia
- New Zealand
- United Kingdom
- South Africa
- Japan
- China
- Philippines
- Other (please specify) _____________________

**2.5 What is you weekly combined household income**

- Negative income / Nil income
- Less than $299
- $300 - $399
- $400 - $599
- $600 - $799
- $800 - $999
- $1000 - $1249
- $1250 - $1499
- $1500 - $1999
- $2000 - $2499
- $2500 - $2999
- $3000 - $3499
- $3500 - $3999
- $4000 – $4999
- $5000 or more
- I would prefer not to answer

**2.6 Drug use**

**BEFORE YOU WERE PREGNANT how often did you use any prescribed, non-prescribed or herbal drugs?**

- Never
- Monthly or less
- 2-4 times a month
- 2-3 times a week
- 4 or more times a week

**2.7 Tobacco use**

**Are you currently smoking tobacco cigarettes?**

- I have never smoked
- I smoke daily, about the same as before I was pregnant
- I smoke daily now, but I’ve cut down since finding out I was pregnant
- I smoke every once in a while
- I quit smoking since finding out I was pregnant
- I wasn’t smoking around the time I found out I was pregnant – I had smoked within the last 12 months
- Previous smoker, more than 12 months ago

**2.8 Have you given birth before? This includes both vaginal births and caesarean sections. Please do not count miscarriages or births that happened before 20 weeks (5 months) of pregnancy.**

- No
- Yes

**2.9** **How many children have you given birth to ………………….**

**The following questions relate to your *current* pregnancy**

**3.1 BEFORE you got pregnant, did a doctor, midwife, nurse, or other health worker tell you that you had any of the following health conditions? Please mark all that apply.**

- None
- Diabetes
- High blood pressure or hypertension
- A mental health disorder such as depression, anxiety bipolar disorder or schizophrenia

**3.2 Are you pregnant with:**

- One baby
- Two babies (Twins)
- Three or more babies

**3.3 If you have been pregnant before, have you experienced any of the following in previous pregnancies? Please mark all that apply**.

- This is my first pregnancy
- A preterm baby born before 37 weeks
- Bleeding so much during pregnancy, birth, or after giving birth that you needed a blood transfusion
- A caesarean section
- Loss of a pregnancy after 20 weeks (5 months)

**3.4 Have you had any recent stressors, changes or losses in the last 12 months?**

**E.g. separation, domestic violence, job loss, bereavement, etc**

- Yes
- No

*If yes, please specify*…………………………………………..

**3.5 Is this a planned pregnancy**

- Yes
- No

The full ICHOM Data Collection Reference Guide can be accessed at <https://www.ichom.org/portfolio/pregnancy-and-childbirth/>

**Now we will ask questions about your general wellbeing in the past few weeks**

**4** **In the past FEW WEEKS ……**

| \|  \|  \| Excellent \| Very good \| Good \| Fair \| Poor \| \| --- \| --- \| --- \| --- \| --- \| --- \| --- \| \| 1. 4.1 \| In general, would you say your health is…… \|  \|  \|  \|  \|  \| \| 1. 4.2 \| In general, would you say your quality of life is……. \|  \|  \|  \|  \|  \| \| 4.3 \| In general, how would you rate your physical health? \|  \|  \|  \|  \|  \| \| 4.4 \| In general, how would you rate your mental health, including your mood and your ability to think? \|  \|  \|  \|  \|  \| \| 4.5 \| In general, how would you rate your satisfaction with your social activities and relationships? \|  \|  \|  \|  \|  \| \| 4.6 \| In general, please rate how well you carry out your usual social activities and roles. (This includes activities at home, at work and in your community, and responsibilities as a parent, child, spouse, employee, friend, etc). \|  \|  \|  \|  \|  \| \|  \|  \| **Completely** \| **Mostly** \| **Moderately** \| **A little** \| **Not at all** \| \| 4.7 \| To what extent are you able to carry out your everyday physical activities such as walking, climbing stairs, carrying groceries, or moving a chair? \|  \|  \|  \|  \|  \| \|  \| **In the past 7 days……** \| **Never** \| **Rarely** \| **Sometimes** \| **Often** \| **Always** \| \| 4.8 \| How often have you been bothered by emotional problems such as feeling anxious, depressed or irritable? \|  \|  \|  \|  \|  \| \|  \| **In the past 7 days…..** \| **None** \| **Mild** \| **Moderate** \| **Severe** \| **Very severe** \| \| 4.9 \| How would you rate your fatigue on average? \|  \|  \|  \|  \|  \| |
| --- | --- | --- | --- | --- | --- | --- | --- | --- | --- | --- | --- | --- | --- | --- | --- | --- | --- | --- | --- | --- | --- | --- | --- | --- | --- | --- | --- | --- | --- | --- | --- | --- | --- | --- | --- | --- | --- | --- | --- | --- | --- | --- | --- | --- | --- | --- | --- | --- | --- | --- | --- | --- | --- | --- | --- | --- | --- | --- | --- | --- | --- | --- | --- | --- | --- | --- | --- | --- | --- | --- | --- | --- | --- | --- | --- | --- | --- | --- | --- | --- | --- | --- | --- | --- | --- | --- | --- | --- | --- | --- | --- |

**4.10 How would you rate your pain on average? (Please circle one)**

| 0 | 1 | 2 | 3 | 4 | 5 | 6 | 7 | 8 | 9 | 10 |
| --- | --- | --- | --- | --- | --- | --- | --- | --- | --- | --- |

**No Pain Worst pain imaginable**
